# SARS-CoV-2 Variants of Concern and Variations within Their Genome Architecture: Does Nucleotide Distribution and Mutation Rate Alter the Functionality and Evolution of the Virus?

**DOI:** 10.3390/v14112499

**Published:** 2022-11-11

**Authors:** Varsha Ravi, Aparna Swaminathan, Sunita Yadav, Hemant Arya, Rajesh Pandey

**Affiliations:** 1 INtegrative GENomics of HOst-PathogEn (INGEN-HOPE) Laboratory, Division of Immunology and Infectious Disease Biology, CSIR-Institute of Genomics and Integrative Biology (CSIR-IGIB), Mall Road, Delhi 110007, India; 2 Academy of Scientific and Innovative Research (AcSIR), Ghaziabad 201002, India

**Keywords:** molecular modeling, mutation analysis, nucleotide diversity, RNA secondary structure, VOCs

## Abstract

SARS-CoV-2 virus pathogenicity and transmissibility are correlated with the mutations acquired over time, giving rise to variants of concern (VOCs). Mutations can significantly influence the genetic make-up of the virus. Herein, we analyzed the SARS-CoV-2 genomes and sub-genomic nucleotide composition in relation to the mutation rate. Nucleotide percentage distributions of 1397 in-house-sequenced SARS-CoV-2 genomes were enumerated, and comparative analyses (i) within the VOCs and of (ii) recovered and mortality patients were performed. Fisher’s test was carried out to highlight the significant mutations, followed by RNA secondary structure prediction and protein modeling for their functional impacts. Subsequently, a uniform dinucleotide composition of AT and GC was found across study cohorts. Notably, the N gene was observed to have a high GC percentage coupled with a relatively higher mutation rate. Functional analysis demonstrated the N gene mutations, C29144T and G29332T, to induce structural changes at the RNA level. Protein secondary structure prediction with N gene missense mutations revealed a differential composition of alpha helices, beta sheets, and coils, whereas the tertiary structure displayed no significant changes. Additionally, the N gene CTD region displayed no mutations. The analysis highlighted the importance of N protein in viral evolution with CTD as a possible target for antiviral drugs.

## 1. Introduction

The origin of new RNA viral quasispecies pathogenic to humans, particularly severe acute respiratory syndrome coronavirus 2 (SARS-CoV-2), has made the research community come together and put massive collective efforts towards a better understanding of the viral RNA genome dynamics [1]. Despite great heterogeneity displayed by the RNA viruses within their population, higher mutation rates are not always reflected in the rapid evolution. There are conditions in which viruses replicate efficiently and yet accumulate a few viable mutations through which they can attain genome stability [2]. For instance, the 1918 H1N1 virus had evolved to suppress the CpG presence in their genome, showing a positive selection for the UpA motifs. Later, it was found that the CpG motifs can potentially trigger an antiviral response through interaction with the pattern recognition receptors (PRRs). Therefore, the selective reduction in the CpG motifs could be a viral strategy to escape the immune recognition by the host. Thus, it is important to investigate, understand, and elucidate the driving force of RNA virus heterogeneity caused by the mutations that could alter the viral genetic content [3,4]. Hence, it is vital to address questions such as what configuration of A, T/U, G, and C makes the viral genome stable? Does it vary among the different variants of a virus? What is the characteristic pattern of mutations that drives the viral RNA genome plasticity? Are these findings important for other RNA virus infections for disease severity?

The corona virus disease 2019 (COVID-19) outbreak caused by SARS-CoV-2 has been one of the most rogue pandemics in modern times, which led to unexpected infections and mortality worldwide [5]. However, the number of infected cases and mortality rates vary from country to country [6]. It is important to note that the first available genome sequence of SARS-CoV-2 placed it in the *Sarbecovirus* subgenus of the *Coronaviridae* [7]. With the genome sequence availability, it enabled an immediate analysis of its ancestry, and consequently, Zhou et al. reported a probable bat origin for the COVID-19 outbreak due to the high genetic relatedness of SARS-CoV-2 to RaTG13 [8]. Later, Makarenkov et.al suggested that the SARS-CoV-2 was a chimera of RaTG13 and pangolin coronaviruses, which reasserted that the pangolins might be an intermediate host of SARS-CoV-2 [9]. Moreover, in addition to the suggested zoonotic origin of SARS-CoV-2, several scientists proposed a potential laboratory origin of COVID-19 [10]. Although the zoonotic origin hypothesis is considered more likely, it is still not clearly defined. Hence, it is significant to study the SARS-CoV-2 genome variations due to mutations, which may help us to understand the origin of the virus. 

Fortunately, worldwide sequencing and tracking of SARS-CoV-2 during the current pandemic have given us the opportunity to delve deeper into the RNA genome architecture of the virus. Consequently, the emergence of SARS-CoV-2 variants of concern (VOCs) has led to the natural selection of mutations with distinct functional consequences, which has globally augmented the propagation of the virus over the course of time [11,12]. Subsequently, the Delta variant (B.1.617.2) was observed to be more contagious, with heightened reinfections and mortality rates [13]. Furthermore, the Omicron variant was often attributed to causing a milder disease manifestation [14], but it simultaneously led to increased cases of hospital admissions in children compared to the previous waves of infections [15]. Recently, a study by Saifi et.al highlighted that the mutations associated with COVID-19 mortality patients potentially reduced the drug-binding efficiency of remdesivir in comparison with the recovered of both Delta and Omicron variant-infected patients [16]. Additionally, it is important to note that despite mass immunization of populations against the virus at an unprecedented rate, several vaccine breakthrough infection cases are being reported globally. Consequently, the spike mutations L425R,Y453F [17], and N439K [18] in the receptor-binding motif have been shown to evade the human host immune response and potentially reduce antibody neutralization.

Although intensive efforts have been made to study the spike region dynamics, the mutations outside of the spike region are also likely to contribute to the viral evolution and adaptation. In this regard, a study by Thorne et. al. reported that the Alpha variant has increased sub-genomic RNA and protein levels of the N, ORF9b, and ORF6—the genes which are known to facilitate the virus escaping the human host’s innate immune response [19]. Therefore, it is essential (i) to understand the seemingly independent but interrelated genome architecture of the SARS-CoV-2 VOCs; (ii) study the evolution of the RNA genomes, which contributes to differential mutation rates; and (iii) perform evaluation of disease severity caused by different variants, which can help in understanding the variants’ impacts on public health and decision making [20].

To gain insights into the viral genome architecture that can differentially distinguish the SARS-CoV-2 VOCs combined with their clinical outcomes, we comprehensively analyzed the distinct nucleotide composition that provides stability to the genome and alters the transmission rate and disease severity over the course of time. Herein, we enumerated the AT and GC composition of the SARS-CoV-2 genome at the lineage level—Alpha, Delta, and Omicron; and for the clinical outcomes—Delta recovered (DR), Delta mortality (DM), Omicron recovered (OR), and Omicron mortality (OM). Subsequently, we investigated the mutation profile of these groups and integrated it with the nucleotide composition of the SARS-CoV-2 genome as well as different genomic regions. Further, two-tailed Fisher’s test was performed to highlight the statistically significant mutations, and their functional impacts were elucidated through RNA secondary structure prediction and protein modeling. The findings highlighted the importance of the SARS-CoV-2 N gene that could serve as a potential target for antiviral drugs, which can aid us in better management of COVID-19 with future VOCs on the anvil. 

## 2. Materials and Methods

### 2.1. Genome Sequencing of SARS-CoV-2

Whole genome sequencing of 1397 SARS-CoV-2 samples was performed using the Oxford Nanopore Technology (ONT, Oxford, UK) and Illumina sequencing platforms (Illumina, San Diego, CA, USA). 

#### 2.1.1. Nanopore Sequencing

A total of 310 samples were sequenced using ONT library preparation protocol—PCR tiling of SARS-CoV-2 virus with rapid barcoding (version: PCTR_9125_v110_revB_24Mar2021, ONT, Oxford, UK). In brief, 50 ng of total RNA was taken to synthesize single-stranded cDNA using LunaScript RT SuperMix (New England Biolabs, Cat. No. E3010L, Ipswich, MA, USA)). The cDNA–RNA hybrid was used to amplify the SARS-CoV-2 genome with its specific primer sets (IDT product number: 10007184) and Q5^®^ High-Fidelity 2X master mix (New England Biolabs, Cat. No. M0494S). For sequencing library preparation, the amplified products were ligated with rapid barcode sequences (SQK-RBK110.96) followed by SPRI bead purification. The purified library was then ligated with an adapter protein and loaded on the MinION Mk1B or MinION Mk1C platform.

#### 2.1.2. Illumina Sequencing

Sequencing library preparation was performed using Illumina COVIDSeq for the 1087 samples (Cat. No. 20043675 and reference guide: 1000000126053 v04). The total RNA was utilized to synthesize cDNA, and the viral genome was further amplified using two separate PCR reactions. The pooled amplicons then underwent tagmentation to fragment and tag amplicons with adapter sequences. This was followed by post-tagmentation cleanup, a second round of PCR amplification, and ligation of index adapters. The indexed amplicons were pooled and cleaned using the purification beads. The pooled library was then quantified using Qubit dsDNA HS Assay kit (Cat. No. Q32854). A loading concentration of 11 pM was prepared by denaturing and diluting the libraries in accordance with the MiSeq System Denature and Dilute Libraries Guide (Illumina, Document no. 15039740 v10). Sequencing was performed on the MiSeq system using the MiSeq Reagent Kit v3 (150 cycles) and 2 × 75 bps read length.

### 2.2. Sequencing Data Analysis

#### 2.2.1. Nanopore Sequencing

The ARTIC end-to-end pipeline [21] (2021) was used for the analysis of MinION raw fast5 files up to the variant calling. Raw fast5 files of the samples were base-called and demultiplexed using Guppy basecaller, which uses the base-calling algorithms of Oxford Nanopore Technologies with a Phred quality cutoff score > 7 on a GPU-Linux accelerated computing machine. Reads with a Phred quality score of less than 7 were discarded to filter the low-quality reads. The resultant demultiplexed fastq files were normalized by a read length of 1200 (approximate size of amplicons) for further downstream analysis and aligned to the SARS-CoV-2 reference (MN908947.3) using the aligner Minimap2 v2.17 [22]. Nanopolish was used to index the raw fast5 files for variant calling from the minimap output files. To create the consensus fasta, bcftools v1.8 [23] was used over the normalized minimap2 output.

#### 2.2.2. Illumina Sequencing

Fastqc [24] was performed for all the raw fastq files generated from the Illumina sequencing in order to check the Phred quality scores of all the sequences. A Phred quality score threshold of >20 was used for filtering reads from all the samples. Subsequently, adapter trimming was performed using the Trim Galore tool [25], and alignment of the sequences with the SARS-CoV-2 genome was performed using the HISAT2 algorithm [26,27]. BEDTools was used to generate the consensus fasta using the unaligned/filtered reads, and variant calling was performed using the high-quality reads. The sequencing depth and genome coverage for all the samples are available in the Table S1.

### 2.3. Data Collection and Patient Categorization

A total of 1397 SARS-CoV-2 positive nasopharyngeal RNA samples included in the study were anonymized. Moreover, all the samples had ≥80% genome coverage and an average sequencing depth of >100X (Supplementary File S1: Table S1). Further, the samples were stratified at two levels based on the lineage infected and the clinical outcome of the COVID-19 patients, recovered and mortality. Random sampling was performed at the second level of classification using python packages to avoid any sample size bias.

### 2.4. Nucleotide Composition Analysis

The percentage distribution of A, T/U, G, and C were calculated using the aligned Fasta sequence of the study groups: (i) VOCs—Alpha, Delta, and Omicron, and (ii) Delta recovered (DR), Delta mortality (DM), Omicron recovered (OR), and Omicron mortality (OM). Furthermore, the distributions of AT/U and GC dinucleotides were also calculated for the sample cohorts. All the calculations were performed for the whole genome of SARS-CoV-2 as well as for the different genomic regions within the SARS-CoV-2 genome (Supplementary File S2: Table S2). 

### 2.5. Mutation and Statistical Analysis

To obtain the mutation spectra of our cohort, the VCFs of Alpha, Delta, and Omicron as well as DR, DM, OR, and OM subgroups were merged separately using bcftools [23]. Subsequently, the relative frequency of each mutation was calculated (Supplementary File S3: Table S3). To highlight the statistically significant mutations, Fisher’s exact test was performed for lineage-based and clinical-outcome-based groups separately using python programming. Furthermore, the Phi correlation coefficient test using R programming was carried out to examine the associations of the significant mutations with the clinical outcomes—recovered and mortality (Supplementary File S4: Table S4).

### 2.6. RNA Secondary Structure Prediction

To elucidate the functional consequence of mutations, RNA secondary structure prediction was performed using the RNAfold program [28], one of the core programs of the Vienna RNA package that can predict the minimum free energy (MFE) using the dynamic programming algorithm [29]. Selective statistically significant synonymous mutations were used to determine their impact on RNA structure as compared to the wild-type SARS-CoV-2 by taking the 250 nucleotides upstream and downstream of the mutation sites. The minimum free energy (MFE) was obtained for both wild type and mutant, which is an important indicator to highlight whether the mutations affect the folding stability of the respective RNA structure (Supplementary File S5: Table S5).

### 2.7. Molecular Modeling and Optimization

To determine the effects of mutations on the protein structure, molecular modeling was performed for selected significant mutations. We retracted the protein Fasta sequence from Uniprot (accession id: P0DTC9.1) and modeled the protein using the Phyre Server [30]. The modeled structure was further optimized using the WHAT IF web interface [31] and validated through Ramachandran plot analysis (Supplementary File S6: Table S6). Subsequently, the modeled protein was used for secondary structure prediction using the PDBsum online server [32]. Key mutations were incorporated in the modeled protein using PyMOL software [33]. Energy minimization is a key computational step for obtaining a minimized stable structure of the modeled protein. In this study, GROMACS software [34] was used to minimize the wild-type and mutated proteins. AMBER force field [35] was used to optimize the geometry of the modeled/generated N proteins, and the steepest descent method was used for energy minimization.

## 3. Results

### 3.1. Sample Segregation

**Figure 1:**
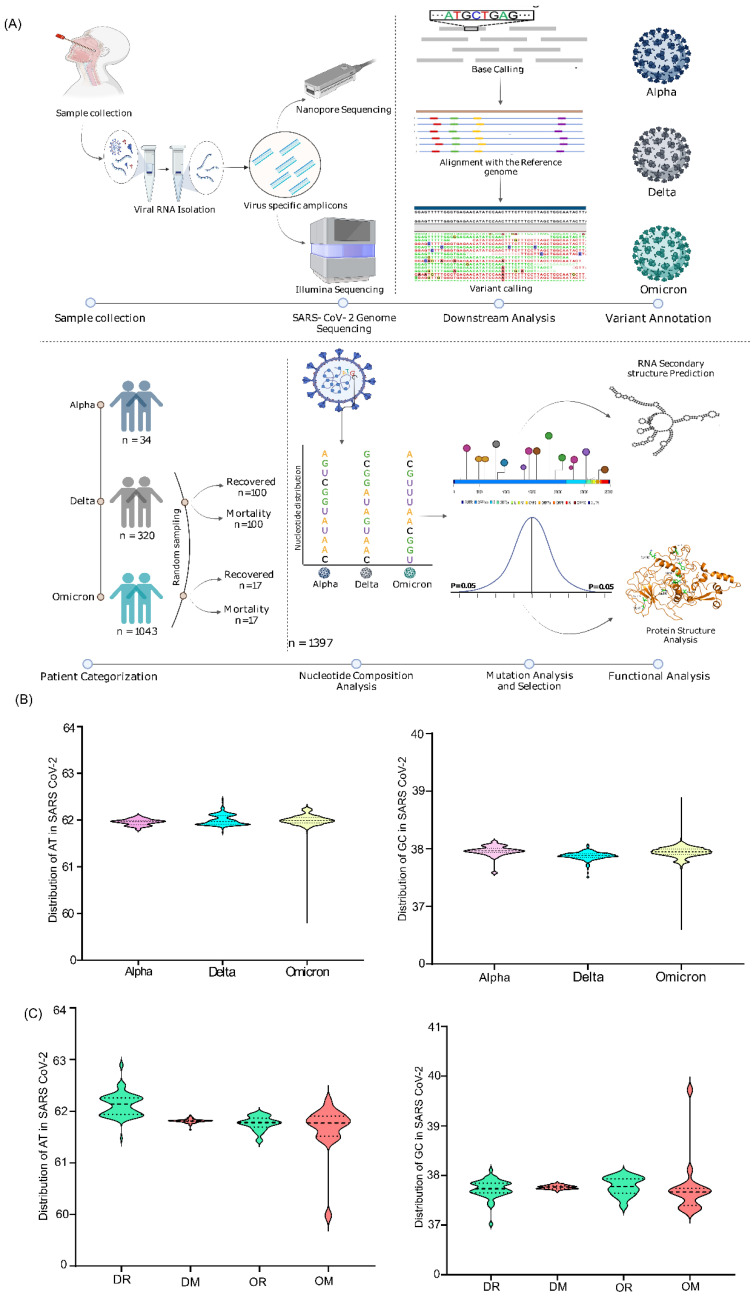
Study design and the Dinucleotide distribution across VOCs at the lineage and clinical outcome level. (**A**) the steps highlight the sample distribution across Alpha, Delta, and Omicron, sequencing data processing, and downstream analysis of the significant mutations. (**B**) AT/U and GC distribution in the genomes of the Alpha, Delta, and Omicron samples included in the study. (**C**) AT/U and GC distribution across the clinical outcomes with VOCs infection—DR, DM, OR, and OM.

The lineage-level stratification of our cohort (n = 1397) yielded a total of three groups—Alpha (n = 34), Delta (n = 320), and Omicron (n = 1043). Subsequently, the secondary categorization based on clinical outcome level resulted in four groups—Delta recovered (DR; n = 100), Delta mortality (DM; n = 100), Omicron recovered (OR; n = 17), and Omicron mortality (OM; n = 17) (Supplementary File S1: Table S1). It is important to mention that the selection of 17 OR samples was based on random sampling for clinical outcome-driven stratification taking gender and age into consideration (Figure 1A).

### 3.2. Nucleotide Composition Analysis

#### 3.2.1. Nucleotide Distribution across the Whole Genome

The nucleotide composition analysis performed both at the lineage and outcome levels revealed a uniform distribution of A, T/U, G, and C across all the VOCs groups in comparison with the Wuhan reference genome, with T/U having the highest representation amongst all the nucleotides (~32%) (Supplementary File S2: Table S2), which was consistent with the other reported studies. Further, looking into the dinucleotide distribution, we found a uniform distribution of AT/U (61.9–62%) and GC (35.88–35.96%) within the VOCs and in comparison to the reference Wuhan strain (62.03% and 37.97%, respectively), both at the lineage and outcome levels (Figure 1B,C). Notably, though the nucleotide distribution was uniform and similar, we found a small percentage difference between the AT/GC compositions, which in terms of numbers is a handful of nucleotides that may have functional role across the VOCs, genomic regions, and clinical outcomes (Supplementary File S2). Even the microscale difference in the nucleotide abundance is important, as it could possibly be present in the specific domains of the SARS-CoV-2 genome, which can affect RNA structure and the protein domains.

#### 3.2.2. Nucleotide Distribution across Different Genomic Regions

**Figure 2:**
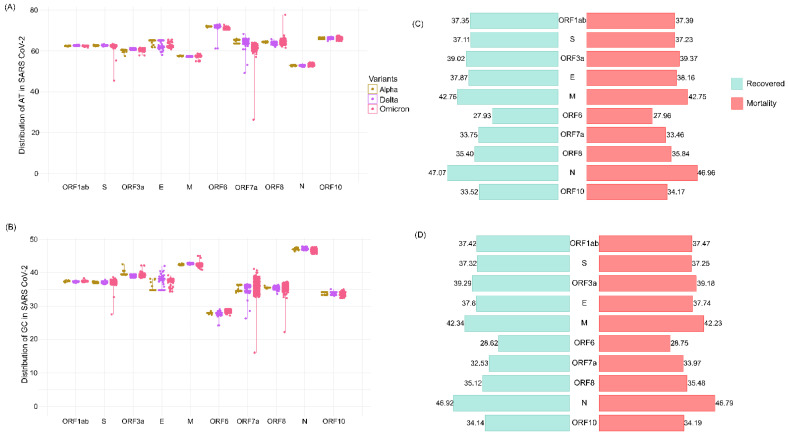
Dinucleotide distribution across the genomic regions of lineage- and clinical-outcome-based sub-groups. (**A**) AT/U percentage across distinct sub-genomic regions of Alpha, Delta, and Omicron. (**B**) GC percentage across different sub-genomic regions of Alpha, Delta, and Omicron. (**C**) Average GC distribution in the clinical outcome groups of Delta—Recovered and Mortality. (**D**) Average GC distribution in the clinical outcome group of Omicron—Recovered and Mortality.

Furthermore, delineating from the whole genome level to different sub-genomic regions of SARS-CoV-2, the analysis highlighted a distinct composition of AT/GC across the genomic regions in the respective groups (Supplementary File S2: Table S2). Intriguingly, we found the structural gene, N, to have the highest GC content across the lineage-based groups, followed by the M and ORF3a genes. On the other hand, ORF6 had the lowest GC percentage in Alpha and Delta, whereas ORF7b was the lowest in Omicron, followed by ORF10 across all the VOCs (Figure 2A,B). The same characteristics were reflected in the recovered and mortality patients of Delta and Omicron (Figure 2C). This possibly highlights the significance of other structural genes during viral evolution in addition to the widely studied genes, ORF1ab and spike. 

### 3.3. Mutation Analysis Revealed High Mutation Rate in the N Gene with Highest GC Percentage

**Figure 3:**
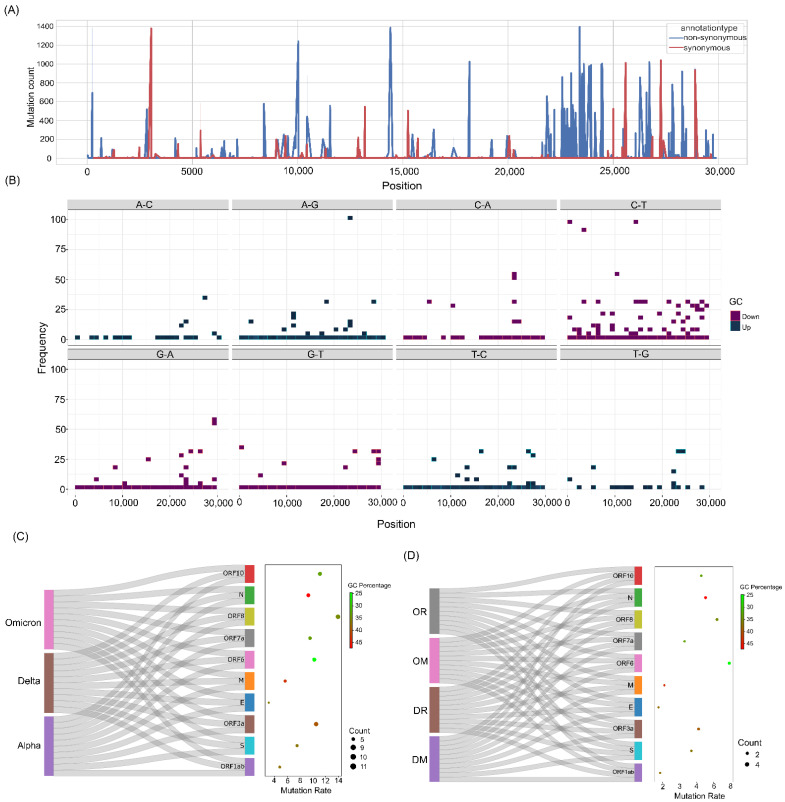
Mutation spectra of the samples and their correlation with GC percentage. (**A**) Synonymous and nonsynonymous mutation distribution across all the samples. (**B**) GC-up and GC-down mutations in the SARS-CoV-2 genomes. (**C**) Correlation of GC percentage and the mutation rate at the lineage level—Alpha, Delta, and Omicron. (**D**) Correlation of GC percentage and mutation rate in the clinical outcome groups—DR, DM, OR, and OM.

To examine the mutational diversity in our cohort, individual mutation analysis was carried out across all 1397 samples. The analysis revealed a total of 1777 mutations, of which 1718 were SNPs, 37 were deletions, and the remaining 3 were insertions (Supplementary File S3). Upon further classification of mutations as synonymous and nonsynonymous, we observed a total of 694 and 950 mutations, respectively (Figure 3A and Supplementary File S3: Table S3). To comprehend the effects of mutations vis-a-vis nucleotide distribution within genomes and the different sub-genomic regions, we classified the variations into (i) mutations that increase the GC content (GC-up) and (ii) decrease the GC content (GC-down). GC-up mutations consisted of A > G, A > C, T > C, and T > G, whereas GC-down mutations were G > A, G > T, C > A, and C > T. Here, we observed an overall higher number of GC-down mutations across the genomes, and notably, the C > T variation was present in a higher number (Figure 3B). Further examination of GC-up and GC-down mutations across the different sub-genomic regions also revealed a dominant representation of GC-down mutations compared to the GC-up mutations. 

Further, to investigate the relative abundance of mutations present in different genomic regions across the lineage- and clinical-outcome-based categories, the mutations were normalized with respect to their corresponding gene length. Subsequently, these genomic regions were correlated with their average GC %age distribution. The analysis revealed two genes, N and ORF3a, to have a higher GC percentage (46.9% and 39.3%) and mutation rate (9.29% and 10.51%), respectively. Strikingly, we observed a very high mutation rate in ORF6 (10.22) even though it had the lowest GC percentage amongst all the sub-genomic regions (Figure 3C). This characteristic mutation pattern was consistent in the genomic regions of outcome-based patient categories—Delta (recovered and mortality) and Omicron (recovered and mortality) (Figure 3D). This seems to highlight that the selection pressure in the coding regions is higher at the amino acid level [36].

### 3.4. Statistically Significant Mutations within the Different Categories

**Figure 4:**
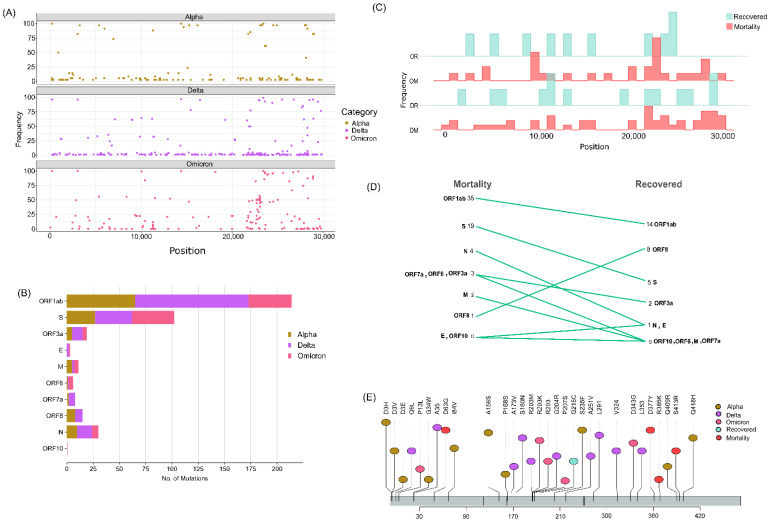
Significant mutations across the study cohorts in the whole genome and different sub-genomic regions. (**A**) Significant mutations associated with Alpha, Delta, and Omicron. (**B**) Mutation count across the sub-genomic regions of SARS-CoV-2. (**C**) Significant mutations associated with the clinical outcome groups of DR, DM, OR, and OM. (**D**) Mutation count in the clinical outcome groups correlated with the distinct genomic regions of SARS-CoV-2. (**E**) Lollipop plot displaying significant mutations of N gene across the study cohorts.

To gain further insights into the mutation associations with different VOCs and clinical outcomes, Fisher’s test was carried out along with Phi coefficient analysis to understand the direction of association of the mutations. Resultantly, we obtained a total of 432 mutations at the lineage level (Figure 4A), where Delta had the highest percentage of mutation distributions at 46%, followed by Alpha at 29.6% and Omicron at 24.3% (Figure 4B and Supplementary File S4: Table S4). Moreover, a higher number of mutations was predominantly seen in the ORF1ab, spike, and nucleocapsid region overall as well as within each VOC—Alpha, Delta, and Omicron. Herein, notably, we observed that three mutations, G28881A, G28882A, and G28883C, in the N gene co-occurred and were present at a high frequency in Alpha and Omicron, whereas they were missing from the Delta lineage. Though these mutations were seen in Alpha and Omicron at a considerable frequency, they were significantly associated with the Omicron lineage (*p* value = 7.7 × 10^−225^; 6.41 × 10^−202^; 1.47 × 10^−204^). Interestingly, we also observed the occurrence of two different mutations at the same position 28881 in the N gene, where G28881A was significantly associated with Omicron (*p* value = 7.7 × 10^−225^) and G28881T was significantly associated with Delta (*p* value = 1.53 × 10^−271^). 

Although the mutation G28881A was present in Alpha at a high frequency (82.35%), it is interesting to note that it became a clade-defining mutation in the Omicron lineage with an observed mutation skip in Delta during its evolution. In contrast, the frequency of the mutation G28881T was much higher (96.25%) in Delta, whereas it was zero in Alpha and 1.24% in Omicron, thus showing a significant predominant association with the Delta lineage only. Additionally, we also noticed the co-occurrence of three other mutations—G28280C, A28281T, and T28282A—with equal high-frequency presence (91.17%) in Alpha but which became deselected in Delta and Omicron during their evolution. 

Fisher’s test performed at the outcome level in the Delta cohort revealed a total of 61 mutations, of which 14 mutations were significantly associated with the recovered group and 47 mutations were significantly associated with the mortality group. On the other hand, we observed 10 mutations in the Omicron recovered and 31 mutations in the Omicron mortality group (Figure 4C). Intriguingly, a higher number of mutations was associated with mortality cases in both Delta and the Omicron (Figure 4D). Additionally, the genomic regions of ORF1ab, spike, and N genes were observed to have a higher number of mutations. With the highest GC percentage and a high mutation rate in the N gene, the mutations significantly associated with the N gene were further delineated to study the functional impacts on the RNA structure and the protein. Figure 4D depicts the significant mutations in the N gene. 

### 3.5. RNA Secondary Structure Modulated by the Specific Mutations

**Figure 5:**
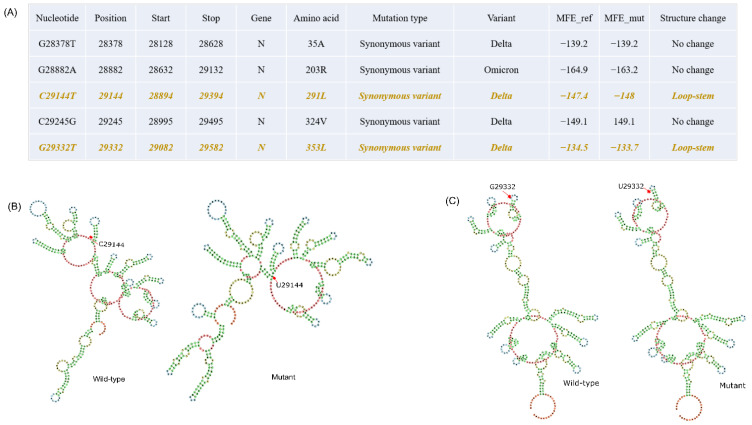
Synonymous mutations in the N gene and their effect on the RNA structural changes. (**A**) Table depicting the synonymous mutations of the N gene with two mutations (Orange color text) reflecting the RNA structure modulation. (**B**) RNA secondary structure of C29144T mutation, the wild type and the mutant with red arrows depicting the site of mutation. (**C**) RNA secondary structure of G29332T mutation, the wild type and the mutant with red arrows depicting the site of mutation.

To elucidate the impact of mutations on the RNA secondary structure, we selected five synonymous mutations from the N gene (Figure 5A). While the mutation G28882A was significantly associated with Omicron, the rest of the four mutations were significantly associated with Delta (Figure 5A). From the analysis, we found that the RNA secondary structure is changed when C to U and G to U occur at positions 29144 and 29332, respectively. Subsequently, we observed that both G29332U mutations formed a stem instead of hairpin loop in the mutant with distinct minimum free energy (MFE) (Figure 5). Intriguingly, the mutation C29144U resulted in more negative MFE, which enhanced the RNA thermodynamic stability, while the mutation G29332U gave rise to less negative MFE, which showed a destabilizing effect on the RNA (Figure 5A). Additionally, secondary structure prediction for all the N gene synonymous mutations was performed, and MFEs were observed in the RNA secondary structures (Supplementary File S5: Table S5).

### 3.6. Structural Modification in the N Protein

**Figure 6:**
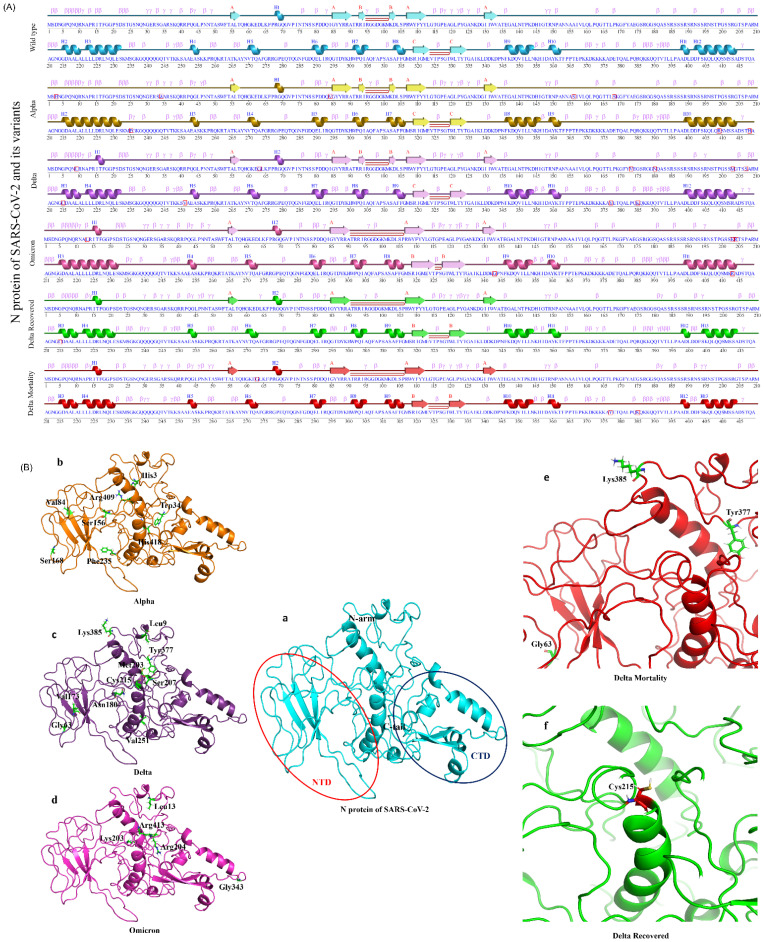
2D and 3D structural changes in the N protein with respect to the variants and their clinical outcomes. (**A**) Secondary structure representation of the targeted N protein and its variants, along with the “□” represents the mutated amino acid(s). (**B**) Molecular modeling of the N protein wild type (**a**) (Red circle represents the NTD domain and the dark blue color represents the CTD domain), Alpha (**b**), Delta (**c**), Omicron (**d**), DM (**e**), and DR (**f**).

The statistically significant missense mutations from the N gene of the lineage- and the outcome-level groups (Table 1) were used for protein secondary structure prediction, using PDBsum with the default parameters. Notably, there were no significant N gene mutations in the OR group. The result showed that the wild-type N protein was made up of 16.7% helix, 7.9% beta-sheet, and 75.4% coil region. Similarly, the secondary structures of the other variants (Alpha, Delta, Omicron, DR, DM, and OM) were observed in the ranges of 16.7–18.1% (helix), 6.9–7.9% (beta-sheet), and 74–75.8% (coil region) (Figure 6A and Table 1). To understand a protein’s biological function, the three-dimensional structure (arrangement of atoms of amino acids in 3D) plays an important role. Further, for tertiary structure prediction, the mutations were incorporated into the structure of the N protein (wild type), which generated six structures of different variants (Figure 6B). The structures were validated via Ramachandran plots (Supplementary File S6: Table S6) and were found to have more than 97% residues in the allowed regions. After validation, energy minimization (using GROMACS) of the modeled and the generated structures was performed and achieved the most stable conformation of the protein. The minimized N protein structures (variants) were superimposed with the wild type and were found to have RMSD in the range of 0.066–0.209 Å, which suggests that wild-type and the variants of N protein are structurally similar.

### 3.7. Validation of the Findings in Independent Cohorts

We carried out the same analysis using two datasets of different origins, (i) Indian but non-CSIR-IGIB, with India being a very vast country with a diverse population and geographical regions, and (ii) from the USA. A total of 1007 SARS-CoV-2 genome sequence data were retrieved from GISAID with a filter of the mentioned origins (500 sequences from India and 507 from the USA) and the timeline of December 2021 to February 2022 (Supplementary File S8: Table S8). Since our study focused on only the protein-coding genes, the same was considered to interpret the results of the USA and the Indian origin samples. 

The analysis across the USA sample cohort showed the N gene to have the highest GC% (46.97%) followed by the M (42%) and ORF3a (39.32%) genes. Subsequently, the normalized mutation rate comparison analysis showed the ORF3a to have a 14.86% mutation rate, followed by the N (8.41%) and the M (6.58%) genes. A similar trend was observed in the Indian sample cohort as well, where the N gene had the highest GC content of 47.01%, followed by the M (42%) and ORF3a (39.27%) genes (Supplementary File S9: Table S9). The mutation analysis revealed a higher mutation rate in the M gene with 7.32%, followed by the N and ORF3a (6.98 and 6.76%) genes, respectively (Supplementary File S10: Table S10a–d). 

Combinedly, correlating the GC% of the respective genes and the normalized mutation rates, the N gene shows the highest GC content coupled with a high mutation rate in the SARS-CoV-2 genomes, which is consistent with the findings drawn from our in-house sample dataset of 1397 samples. Supplementary File S8 contains the metadata, Supplementary File S9 shows the average AT and GC percentage of the sample cohorts, and Supplementary File S10 shows the mutations with their normalized rates.

## 4. Discussion

RNA viruses exist as dynamic and diverse populations shaped by constant mutation(s) and selection for better adaptability to different micro-environments in the multitude of hosts [37]. One such RNA virus, SARS-CoV-2, caused the worldwide COVID-19 pandemic and has had devastating impacts on public health and the economy. At the same time, undoubtedly, the control measures that were taken to contain the infection, with vaccine development being the primary measure to immunize the population against the virus, progressed at an unprecedented rate as an immediate response to COVID-19. [38]. Yet, several vaccination breakthrough cases were reported and became less common with time globally, which potentially raises questions about the efficacy of the developed vaccines [39,40,41,42]. This reinforces the need to explore the multi-dimensionalities of the underlying observation, wherein it is imperative to study the pathogen characteristics leading to different disease trajectories [43,44,45]. With the emergence of several variants of SARS-CoV-2, which led to a big surge of infections in different populations, the central question concerns whether the viral sequences have evolved to differentially optimize genome stability through mutations that could affect the genome nucleotide composition or vice versa [1]. To gain insights into this issue, we comprehensively studied if there could be a differential nucleotide composition that provides stability to the genome to alter the transmission rate and disease severity over the course of time. 

The nucleotide composition analysis across all the VOC groups at the lineage and outcome level demonstrated a higher presence of the nucleotide U, which is consistent with other reported studies [46,47,48]. This could be a result of natural selection because higher U content and smaller genome size can make the virus replicate more efficiently. Moreover, less host energy is required to disrupt the viral RNA secondary structures with relatively higher U content, which can make the virus more infectious, thus essentially increasing the transmission rate [49]. Consequently, we also observed that the mutations C > T and G > T were much higher in number, reflecting the SARS-CoV-2 genome bias towards a higher U content. Particularly, the C > T variation was observed in the highest number among all the study samples, possibly because C to T changes require a mere deamination of the C nucleotide. Consequently, the lower abundance of the GC dinucleotide has been widely seen in SARS-CoV-2 virus genomes and is also correlated with its moderate virulence as compared to MERS-CoV and SARS-CoV-1 [46]. Though the comparative nucleotide composition analysis demonstrated an average minimal sequence divergence across the VOC groups at the lineage and clinical outcome levels, it is imperative to explore the functional importance of the differential nucleotide numbers seen across the same groups. 

Upon examining the nucleotide diversity across the distinct SARS-CoV-2 sub-genomic regions, the N gene showed the highest GC percent of around 47%, which is higher than the average GC percentage of the entire genome. The N gene is reported to be one of the important structural proteins in a virus particle that can modulate the genome transcription and the virulence [50]. Moreover, the SARS-CoV-2 N gene was observed to be highly conserved [51]. Interestingly, in our study, we found the N gene to have a higher mutation ratio after the ORF3a gene, combined with the highest GC percentage across all the groups. Furthermore, Fisher’s test was performed to see the statistical significance of these mutations, which also revealed a high number of mutations in the N region of the SARS-CoV-2 genome after ORF1ab and spike—at the lineage level (Alpha, Delta, and Omicron) and the clinical outcome level (Delta—recovered and mortality; Omicron—recovered and mortality). Notably, the N gene mutations that are almost exclusive to Delta—D63G (95.6%) and D377Y (92.05%)—were significantly associated with mortality and were also reported in breakthrough infections [52]. Strikingly, the mutation D63G was reported to be present in the recombinant strain of Delta and Omicron [53].

Elucidating the functional impact of mutations is very important in order to determine the stability of virus propagation over the course of time. In our study, we found that in the N gene, the RNA secondary structure was changed when C to T and G to T mutations occurred at positions 29144 and 29332, respectively. While the mutation C29144U stabilized the RNA structure, G29332U resulted in a comparatively unstable structure with lower negative MFE than the wild type. Interestingly, there is evidence from the previous study that the most frequent mutations to occur in the SARS-CoV-2 genomes are C to T and G to A. These mutations are related to the role of the cell-derived apolipoprotein B mRNA-editing enzyme, which leads to C-to-U deamination of a single cytidine base in the nuclear apoB transcript, introducing a translational termination. Besides that, the G29332U mutation reduced the folding stability of the RNA secondary structure, which could affect the polypeptide translation and folding. The previous studies suggested that stable RNA structures play a key role in reducing the translation speed to prevent “ribosomal traffic jams” so that the newly translated polypeptides can fold properly [54]. 

Furthermore, we studied the N gene missense mutations’ effects on protein stability. SARS-CoV-2’s N protein is a 419 AA, 45.6 kDa, positively charged, unstable hydrophobic, and poorly heat-resistant protein that is essential for virus survival [55]. In this study, the secondary and tertiary structure of the N protein (wild type and mutants) were predicated computationally, and we found that they are structurally similar. Few variations observed in the secondary structures, such as the helix (15–18 AA), were found in Delta, Omicron, DR, DM, and OM, whereas they were absent (converted into coil) in the wild type and Alpha. A beta-sheet structure (93–94 AA) was present in the wild type, Alpha, and Delta, whereas it was missing (converted into coil) in Omicron, DM, DR, and OM. Similarly, the coil region (220–222 AA and 353–356 AA) was converted into a helix in the Alpha variant. Moreover, Alpha, Delta, and Omicron had helix regions at 398–414 AA, whereas the wild type at 401–402 AA and OM, DR, and DM at 401–403 AA had coil regions between the helix structures. In three-dimensional structures, a lower deviation was observed after the superimposition of the variant’s N protein structure with the wild type. Most of the mutations were found in the RNA-binding domain or NTD and intrinsically domain regions (IDRs) of the SARS-CoV-2’s N protein. Interestingly, no mutations were reported in the C-terminal domain except D343G in the Omicron variant, which suggests that CTD of the N protein could be used as a potential drug-binding site. Several researchers reported that the CTD of the N protein plays an essential role in viral RNA binding, packaging of the SARS-CoV-2 viral RNA, and transcriptional regulations [56,57]. A drug repurposing approach or novel lead design technique may be used to identify/design drug molecules for the treatment of COVID-19 by targeting CTD of the N protein for the betterment of the disease. This is especially important as we foresee the continued evolution of the new SARS-CoV-2 sub-lineages with a regional and global footprint.

To understand this further, we additionally carried out recombination analysis for the 1397 whole genome sequences of in-house samples used in our study using RDP4 software (Supplementary File S7: Table S7). Resultantly, a total of two recombinant events were observed in a consensus between at least three detection methods, with 36% of the total genome sequences being detected as recombinant. For these recombination events, we noted the breakpoint positions to be in the 5’ region of the ORF1ab gene. However, it is important to note that the recombinant events observed were flagged by RDP4 as being possibly driven by other processes despite the support of at least three recombination detection methods. Furthermore, the recombinant sequences in the second recombinant event were labeled as possibly misidentified recombinant sequences by RDP4 (Supplementary File S7). It seems promising, but we did not detect any high-confidence recombination signals in the SARS-CoV-2 genomes of the VOCs—Alpha, Delta, and Omicron.

In conclusion, the results reported here show our efforts to comprehensively investigate the viral RNA dynamics vis-a-vis the mutations and their functional impacts on the virus. This can aid us in understanding the emergence and tracking of new variants along with the elucidation of different disease trajectories. Moreover, examining the immune escape mutations could possibly guide us in better designing of vaccinations and antiviral drugs to ameliorate the observed COVID-19 severity. 

## Figures and Tables

**Table 1:** Secondary structure changes in overall N Protein.

N Protein	Mutations Incorporated	Helix (%)	Beta Strand (%)	Coil Region (%)
Wild type	-	16.7	7.9	75.4
Alpha	D3H/V/E, G34W, I84V, A156S, P168S, S235F, Q409R, and Q148H	17.9	7.9	74.2
Delta	Q9L, D63G, A173V, S180N, R203M, P207S, G215C, A251V, D377Y, and R385K	17.4	7.9	74.7
Omicron	P13L, R203K, G204R, D343G, and S413R	18.1	7.9	74
DR	G215C	17.2	6.9	75.8
DM	D63G, D377Y, and R385K	17.2	6.9	75.8
OM	S413R	17.2	6.9	75.8

## Data Availability

The clinical dataset collected and analyzed as part of this study is available in the Supplementary File in Table S1.

## Supplementary Materials

The following supporting information can be downloaded at: https://www.mdpi.com/article/10.3390/v14112499/s1, Table S1: Sample-wise sequence information; Table S2: Average Percentage of AT and GC across sample cohort; Table S3: List of mutations across lineages and clinical outcomes; Table S4: Statistical significance of lineages and outcomes; Table S5: RNA Secondary Structure prediction; Table S6: Protein quality check; Table S7: Recombination analysis: Table S8: Metadata for sample cohort; Table S9: Average Percentage of AT and GC across USA and India (non-IGIB) sample cohort; Tables S10a–d: List of mutations and normalized mutation rates across USA and India cohort.
